# Next generation legged robot locomotion: A review on control techniques

**DOI:** 10.1016/j.heliyon.2024.e37237

**Published:** 2024-09-12

**Authors:** Swapnil Saha Kotha, Nipa Akter, Sarafat Hussain Abhi, Sajal Kumar Das, Md. Robiul Islam, Md. Firoj Ali, Md. Hafiz Ahamed, Md. Manirul Islam, Subrata Kumar Sarker, Md. Faisal Rahman Badal, Prangon Das, Zinat Tasneem, Md. Mehedi Hasan

**Affiliations:** Department of Mechatronics Engineering, Rajshahi University of Engineering & Technology, Rajshahi 6204, Bangladesh

**Keywords:** AI, CoM, Legged robots, Locomotion, Control, Next generation

## Abstract

The next generation of autonomous-legged robots will herald a new era in the fields of manufacturing, healthcare, terrain exploration, and surveillance. We can expect significant progress in a number of industries, including inspection, search and rescue, elderly care, workplace safety, and nuclear decommissioning. Advanced legged robots are built with a state-of-the-art architecture that makes use of stereo vision and inertial measurement data to navigate unfamiliar and challenging terrains. However, designing controllers for these robots is a difficult task due to a number of factors, including dynamic terrains, tracking delays, inaccurate 3D maps, unforeseen events, and sensor calibration issues. To address these challenges, this paper discusses the current methods for controlling autonomous-legged robots. Our primary contribution is comparative research on robot control strategies such as virtual model control (VMC), model predictive control (MPC), and model-free reinforcement learning (RL). This paper provides information on different strategies for controlling autonomous legged robots and discusses the potential advancements and applications of this technology in the future. The aim of this study is to assist future researchers in making informed decisions on the selection of optimal control strategies and innovative concepts when developing and working with legged robots.

## 1 Introduction

Our surrounding terrains are unstructured, harsh, unstable, deformable, and unsafe. In such situations, legged robots have intrinsic movement benefits. They can cross terrain and hurdle barriers, making them ideal for various tasks. These include search and rescue, inspection in crowded and complex areas, planetary exploration, manufacturing, and agriculture [1]. In recent years, numerous legged systems have moved precisely and consistently in controlled environments [2]. However, there are still many obstacles to overcome before they can be widely used in everyday life [3]. They struggle in uncontrolled conditions despite excelling in controlled environments. The prominent challenges of legged robot locomotion control are to locate appropriate places for creating foot contact with the environment and to produce corresponding dynamic movements. Even state-of-the-art legged robots face challenges in adapting to varying terrains, slippery surfaces, negotiating overground obstacles, managing payload and weight distribution, and recovering from stumbles [12], [13]. Also, model predictive control (MPC) methods and numerical trajectory optimization are currently extensively utilized in the broad approach that pervades nearly all forms of legged robot locomotion. The same techniques are being modified for different leg designs and numbers of legs as well as for both flat ground and rough terrain. The energy efficiency is also much inferior compared to that of biological systems. The majority of research on robot locomotion on uneven terrain has centred on software development, including perception approaches. As a result, there is a lack of assessment of the actual locomotion performance in such difficult terrain. This leads to major inaccuracies, resulting in the infamous “reality gap” [14]. In addition, exclusive reliance on state feedback, a lack of sensor-based control, and robust control are significant drawbacks of the currently available research on the control mechanisms of legged locomotion. On this basis, a comparison table of some existing papers is depicted in Table 1 of this paper.

**Table 1:** Comparison of some existing review papers with this article (Parameters: 1. Desired Architecture 2. Smart application 3. Issues and challenges 4. Taxonomy 5. Expected Locomotion Profile 6. Vision-based direction 7. Locomotion generation 8. Controller specification techniques.)

Authors	Year	Objectives of the paper	Contribution	Limitation	1	2	3	4	5	6	7	8
Boussema et al. [4]	2019	To concentrate on the disturbance recovery of the legged robot using a functional impulse set	The action or process of barrier regaining was discussed	Only concentrate solely on the ideas of measured limb powers for online motion development and adaptation				X		X	X	

Raw et al. [5]	2019	To provide a fresh examination into how the limb of quadrupeds morphologies affect them when they perform quick, transitory movements like acceleration and deceleration.	The concept of mechanical engineers creating more nimble quadruped robots in the future was discussed.	Do not focus on applications and open issues.		X	X			X	X	

Thor et al. [6]	2020	To outline a general framework for legged robot locomotion control and a method for control policy optimization	The framework's scalability to be used both as a decentralized controller for specific legs and leg pairs and as a centralized controller for all the legs of a robot was demonstrated	Lacks discussion about complicated surroundings including slopes, stairs, pipelines, and uneven terrain.			X	X		X		

Kim et al. [7]	2020	To develop a novel WBC, known as the whole-body locomotion controller (WBLC), capable of allowing trial dynamic movement on passive-ankle biped robots without help	Several feedback controllers and whole body control formulation were tested in this paper	Having shortcomings in investigating more adaptable walking and turning movements in a congested setting			X	X		X		

Zhongyu Li et al. [8]	2021	To show a model-free reinforcement learning paradigm for creating dependable movement strategies that can be applied in emulation to real upright motion	Domain random sampling is used to allow policies to obtain robust behaviours across variations in system dynamics, easing the move from modelling to reality.	Lacks information about investigation of how cassie the robot might learn more dynamic and agile behaviours by expanding on the methodology depicted		X		X		X		X

B Chong et al. [9]	2022	To present basic movement control structure for multi-legged locomotors.	Combining two methods, the development of a generic-shape management strategy provides basic patterns of self-deformation (or “gaits”) for effective movement in a range of robot shapes.	Only general locomotion structure of control strategies was showed		X				X	X	

Szabo et al. [10]	2023	To walk via genetic algorithms and can self-recover after falling.	By using genetic algorithms to control methods, this study enhances biped robot learning and recovery, providing enhanced stability, flexibility, and resilience in challenging scenarios.	It was shown that stable walking and efficient fall recovery could be achieved only by optimizing control settings or strategies.		X		X		X	X	

Szczecinski et al. [11]	2023	To provide a historical view of how studying insect and arthropod neuro mechanics has inspired the design and control of hexapod robots.	An examination of the historical control methods for hexapod robot walking, current promising approaches, and potential future advancements in this field	It was shown that core pattern generation contributes, but a comprehensive account of locomotion generation is still lacking.	X	X		X	X		X	

Proposed paper	-	To show different types of locomotion control techniques	The architecture and technology at a fundamental level was discussed to bridge the gap between different types of control method	-								

To perform more effectively and intelligently, today's legged robots must evolve [15]. Motivated by the imperative need to advance legged robot technology, this review paper addresses the challenges hindering their integration into diverse and unpredictable environments. These challenges, encompassing variations in terrains, obstacle avoidance, and adaptability to unforeseen conditions, underscore the necessity for robust control mechanisms. The exploration into dynamics, energetics, and system characteristics aims to bridge the current capability gap and envision a future where legged robots navigate any surface seamlessly.

Holmes et al. [16] illustrate that walking on legs is more feasible than swimming or flying. This would make it possible for robots to work the same way in controlled situations and move over rough terrain. Even letting them go to unsupervised remote areas will not hinder them from traversing any surface, leaping barriers, or avoiding footholds. In this regard, Carpentier et al. [17] examined the dynamics and control of legged robots in terms of contact planning and trajectory optimisation. They gave an overview of the standard way to control legged robots, which is to set up a series of interactions with the environment. Kashiri et al. [18] presented an overview of recent advances in the development of energy-efficient robotic systems with legs. They examined a number of robotic actuators that utilise compliance in parallel and series with the drivetrain to enable energy recycling for locomotion. This study provides insight into the principles governing locomotion. It provides further details on models for the dynamics, energetics, and system characteristics. Mahapatra et al. [19] evaluated the effects of walking parameters on energy consumption as well as static or dynamic robot stabilisation during gait generation on various surfaces. Meanwhile, Pardo et al. [20] conducted a comprehensive literature analysis of scientific findings pertaining to legged locomotion on uneven terrain. To use robots in dangerous and unpredictable situations, especially on uneven terrain, they should walk more like humans and animals [21]. They are able to move on various surfaces resembling people or insects as biomimicry. The study of legged robotics, such as bipedal robots, informs the design and operation of assistive devices and applications. They also help study human and animal mobility. Robotic exoskeletons and prosthetic legs can give patients superhuman abilities like walking and running [22]. Numerous studies and reviews have been done, as this is such a promising area for research. Our paper explores the topic of legged robot locomotion, covering various aspects such as locomotion architecture, taxonomy, profiles, vision-based direction, generation of robots, challenges of developing the next generation of legged robots and control techniques. The aim is to enable researchers and practitioners to better understand and apply this knowledge in their work, leading to advancements in the field by discussing fundamental architecture and technology and highlighting available locomotion control techniques. Table 1 fully describes recent reviews in this field and contrasts them with this study.

The remaining content of our paper is structured as follows: Section 2 delineates the methodological approach adopted to review and analyze the existing literature on legged robot locomotion and control techniques. Sections 3 and 4 briefly describe locomotion and various control strategies for legged robots. The generation of legged robots, forefront technologies, and essential criteria for the next generation are described in Sections 5 and 6, respectively. Next-generation legged robots' applications and challenges are covered in Section 7. The road to developing the next generation of legged robots is discussed in Section 8. Subsequently, a conclusion is drawn in the last section.

## 2 Methodological approach

**Figure 1:**
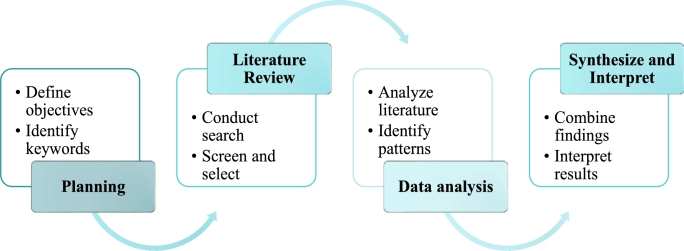
Research methodology in detail.

The research methodology comprises four distinct stages as shown in Fig. 1.

• Planning Stage
  The Planning Stage serves as the foundational phase of the research process. It involves formulating research objectives and delineating key areas of inquiry pertaining to legged robot locomotion and control. To structure the inquiry process, a set of core questions is devised to guide the literature search and review.
  * What are the primary locomotion mechanisms employed by legged robots, and how do they vary across different robot types?
  * How do control algorithms facilitate efficient motion generation and trajectory planning in legged robots?
  * What methodologies are utilised for gait generation and how do they contribute to adaptive locomotion in diverse environments?
  * What role does tail control play in enhancing stability and manoeuvrability in legged robots?
  * What challenges can advanced legged robots address and where can they be applied?
  The formulation of these questions facilitates the identification of relevant keywords and search terms necessary for conducting a comprehensive literature search across reputable academic databases and scholarly repositories.
* Review Stage
  A structured literature search was conducted across reputable academic databases such as IEEE Xplore, ScienceDirect, Springer Link, and Google Scholar. The search strings were formulated using relevant keywords including “legged robot locomotion,” “control techniques,” “vision-based direction,” “gait generation,” and “tail control.” Articles published within the last decade were given priority to ensure the inclusion of recent advances in the field. The review process involved screening titles, abstracts, and full texts to select relevant articles that addressed the key points outlined in the review paper. The selected literature undergoes meticulous scrutiny to ensure alignment with the research objectives and relevance to the thematic focus areas. The screening process encompasses the examination of abstracts, keywords, and content summaries to ascertain the suitability of each document for inclusion in the review.
* Analysis Stage
  The Analysis Stage entails a comprehensive examination and synthesis of the selected literature to distil key insights, trends, and theoretical frameworks related to legged robot locomotion and control techniques. The data extracted are organised thematically to elucidate patterns, challenges, and emerging paradigms within the field. Analytical techniques such as content analysis and pattern analysis are employed to identify recurring themes, theoretical models, and empirical findings in the reviewed literature. Comparative analyses are conducted to evaluate the efficacy and applicability of different locomotion mechanisms, control algorithms, and gait generation techniques in diverse robotic contexts.
* Synthesis and Interpretation Stage
  The synthesis of findings from the planning, review and analysis stages culminates in a comprehensive overview of the state of the art in legged robot locomotion and control. The synthesised insights are interpreted in the context of existing theoretical frameworks, technological advancements, and future research directions within the field. Through a rigorous methodological approach, this research endeavours to contribute to the advancement of knowledge and understanding in the domain of legged robot locomotion and control techniques.

## 3 Locomotion generation and motion control

Legged robot locomotion has evolved significantly, with various designs optimising performance for different environments and tasks. Table 2 provides an overview of these designs, from single-legged hoppers to six-legged hexapods, detailing their mathematical models, parameters, advantages and limitations. This understanding is key to advancing future robots, helping to identify effective stability and adaptation methods for more versatile and resilient designs.

**Table 2:** An overview of many forms of legged robot locomotion.

Locomotion Type	Reference	Mathematical Formulation	Parameters	Usefulness	Applications	Limitations
One legged robot (Hopper)	[23], [24], [25], [26]	Motion's Equation $m\overset{¨}{l}-ml{\overset{˙}{\gamma }}^{2}+$ mgCos(γ)=F $ml^{2}\overset{¨}{\gamma }+2ml\overset{˙}{l}\overset{˙}{\gamma }-$ mlgsin(γ)=τ $\overset{˙}{\Psi }I_{b}=\tau$ Raibert Hopping controller equation: $v_{d}=\frac{\overset{˙}{v}\Gamma_{s}}{2}+k_{\overset{˙}{\nu }}(\overset{˙}{v}-{\overset{˙}{\nu }}_{d})$	I=length of the leg *τ*=torque on the system *γ*=desired angle $I_{b}$=inertia of the body ${\overset{˙}{\nu }}_{d}$=horizontal speed $v_{d}$=controlling desired forward foot position	1. Hop over barriers easily 2. Able to adapt stance without having to be concerned as much about static stability 3. Navigate tough terrain with only one point of contact with the ground 4. No need for leg coordination.	This locomotion mechanism can be employed when the the robot is going in areas where hopping over obstacles is required, such as on stairs, on rocky terrain, and on tiny celestial bodies in deep space.	Has to be blanched, especially in unexpected halting circumstances. State consistency is almost unattainable.

Two legged robot (Biped robot)	[27], [28], [29]	Motion's equation $M(q)\overset{¨}{x}+h\left(\overset{¨}{s},q\right)+\left(\begin{array}{c} 0\\ B \end{array}\right)\tau_{j}$ $+[{}_{J_{\text{Bj}}^{T}}^{Ad_{\text{Hj}}^{T}B}]w_{j}$ High Gain Joint controller equation $\left(\begin{array}{c} MI\;0\\ 0\;M_{\text{ω}\text{B}}\\ . \end{array}\right)\left(\begin{array}{c} {\overset{¨}{r}}_{c}\\ {\overset{˙}{\omega }}_{B}\\ . \end{array}\right)+$ $\left(\begin{array}{c} -mg\\ M_{\omega \text{B}},J{\overset{¨}{q}}_{J}^{d}+h_{\omega_{B}}\\ . \end{array}\right)=$ $\sum_{j}k_{j}\left(\begin{array}{c} I\;0\\ {\overset{˜}{r}}_{\text{cj}}\;I\\ . \end{array}\right)\left(\begin{array}{c} f_{j}\\ \tau_{j}\\ . \end{array}\right)$	$\tau_{j}$=joint torque H=all nonlinear terms B=input mapping matrix $J_{j}$=jacobian's coefficient HjB=$\;\text{A}d_{\text{Hj}}^{T}B$=homogeneous transformation $f_{j}$=contact force $\tau_{j}$=contact torque ${\overset{˜}{r}}_{\text{cj}}$=center of the contact area $w_{j}$=contact wrench $q_{j}^{d}-q_{J}\to 0=joint\;tracking$	1. Compared to multi- legged conditions, the leg condition is simpler 2. Effective for moving across uneven surfaces 3. Establishing stability is faster and simpler with dynamic balancing than with hoppers.	This kind of loco- motion mechanism is often used in human-assisting robots.	When building biped robots, the size and weight of the actuators provide two important challenges. The dynamic balancing is still a problem, however recent study has shown that this problem is generally solvable.

Three legged robot (Tripod robot)	[30], [31]	Equation of motion $M(q)\left(\begin{array}{c} {\overset{¨}{q}}_{a}\\ {\overset{¨}{q}}_{d}\\ . \end{array}\right)+b\left(q,\overset{˙}{q}\right)$ $+\zeta_{c}^{T}F_{c}=\left(\begin{array}{c} \tau_{a}\\ \tau_{p}\\ . \end{array}\right)$	$\tau_{a}$ and $\tau_{p}$=torque $q_{a}=$actuated vector joint $q_{p}=$virtual positive joint $F_{c}=\text{contact}\;\text{force}$ $\zeta_{c}^{T}=$constraint Jacobian associated with the contact wrench	1. Help balance when moving or even standing still. 2. Travel in both circles and routes, depending on the circumstance. 3. Simple to alter the course of their journey.	Due to their superb dynamic balance, these robots may be employed in military applications to carry big loads.	Given how uncommon three-legged mobility is in nature, biological complexity is still in place.

Four legged robot (Quadruped robot)	[32], [33], [34], [34], [35], [36], [37], [38]	Motion's equation ${\overset{˙}{K}}_{G,d}={\bar{I}}_{G}[-k_{p,K}\left(q_{b}-q_{d,b}\right)$ $-k_{D,k}{\overset{˙}{q}}_{b}]$ Control equation $\tau_{S,d}=k_{p,ff\tau_{\text{sff}}}+\tau_{\text{sfd}}$ $\tau_{\text{sfd}}=k_{P,s}\left(\theta_{s}-\theta_{s,d}\right)-$ $k_{D,s}({\overset{˙}{\theta }}_{s}-{\overset{˙}{\theta }}_{s,d})$	$q_{d,b}=$set to zero in straight line ${\bar{I}}_{G}=$motion plan matrix $k_{D,k}=$feedback controller $q_{b}=$footstep position $\tau_{S,d}=$desired torque $\tau_{\text{sff}}=$swing leg inverse dynamics feed-forward term $k_{P,s}>0$, $k_{D,s}>0$ =feedback proportional gain	1. Navigate rocky terrain when climbing, sprinting, walking, and carrying big goods. 2. Have the best dynamics 3. Maintain acceleration and lateral velocity with the assistance of the sensors in the legs, and this aids them in dynamic balancing 4. Support more loads than previous designs.	Due to their ability to move in dangerous human settings and transport goods effortlessly in difficult terrain, they are often utilized in military uses. They may also be created and cared for like pets, serving as a substitute to actual pets.	Compared to other locomotion methods, walking involves a more complicated leg combination.

Six legged robot (Hexapod robot)	[39], [40], [41], [42], [43], [44]	Equation of motion $F_{t}=\left(\begin{array}{c} 0\\ 0\\ -mg \end{array}\right)+R_{b}\sum_{i=1}^{6}leg_{i}F_{i}$ $\times \tau_{T}=R_{b}\sum_{i=1}^{6}leg_{i}\tau_{i}$ $\times {\text{leg}}_{i}=\left\{ \begin{array}{cc} 0\; & \text{leg i is in air}\\ 1\; & \text{leg is in place} \end{array}\right.$	$R_{b}$= orientation and position of body $leg_{i}=$ place flag for leg i $F_{i}=$toe position in weight m=body mass $\tau_{i}=$ bend and hip torque	1.Simulation closely informs the design of the physical machine 2. Employed for stable locomotion using basic clock-driven open-loop controls 3.Designed for stable locomotion tasks due to their static stability.	Replace humans in missions including rescue, reconnaissance, and exploration because of their exceptional dynamic balance.	Walking's complex leg coordination is unusual in nature due to the rarity of six-legged mobility.

### 3.1 The idea of vision-based direction

**Figure 2:**
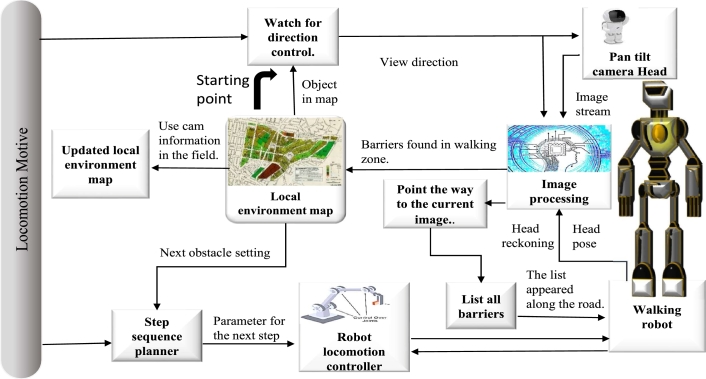
A block schematic of the legged robot's vision-based guiding system.

To choose the appropriate steps toward conquering the challenges, the reactionary step sequential planner needs adequate and exact knowledge about obstacle locations with regard to the robot and obstacle dimensions. The design of the newly developed vision-based guidance system is depicted in Fig. 2. An imaging device mounted on a pan-tilt cranium enables the robot to understand its environment. [45] depicted an image processing system that included a scene analysis module as well as a dynamic computer vision module.

**Figure 3:**
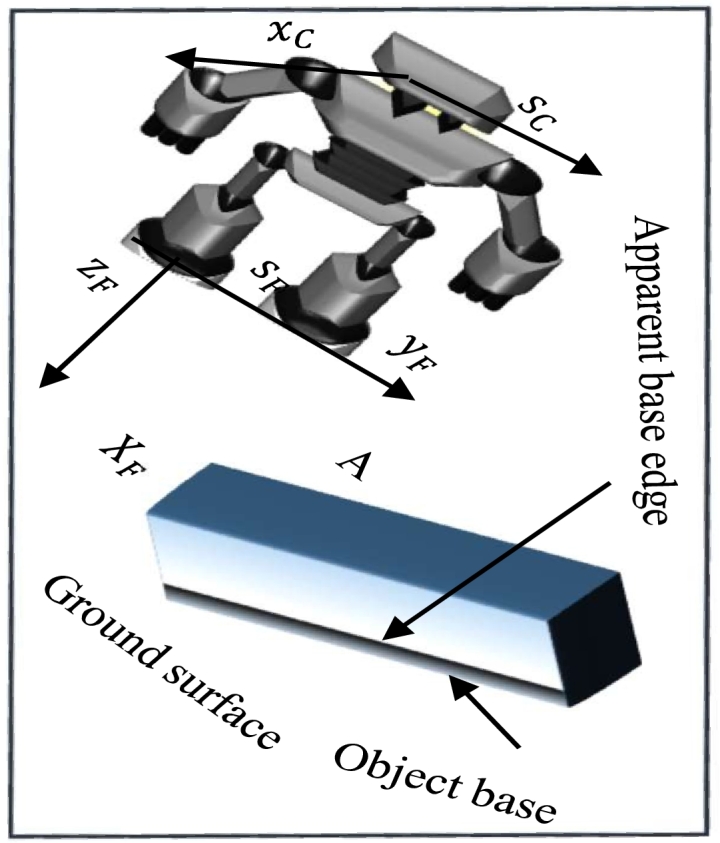
Bipedal vision-guided legged robot with suitable reference frames and a path impediment.

There is just one scene analysis per phase. It comprises determining the walking area's perimeter as well as identifying and categorising any impediments present on the path. The base margins of an obstacle (shown in Fig. 3) are the corners on the contact surface's circumference where the obstruction and the base surface connect. As demonstrated in Fig. 3, the foot support structure, or $S_{F}$, is centred on the sole of the currently standing foot, with the z-axis running counterclockwise to the gravity axis and the x-axis pointing in the direction of walking. This reference frame is appropriate for representing the robot's environment. The robot's foot is assumed to be resting on the ground throughout the execution of a step. This approach allows software modules to reuse the same environmental data at different execution times, as detailed in [46]. The z-coordinate of a point expressed with regard to $S_{F}$ is the height of the point in relation to the floor surface. A real-time feature tracking module can monitor essential object characteristics in real-time, such as the assessment of object bases' discernible edges and close proximity. The scene analysis module can locate objects in a picture. Using the camera's posture, the scene is analysed, and the exact location of an obstacle is determined. $S_{F}$ frames and the difference between these and the FTc using information from the encoders and a representation of a robot's kinematics, two coordinate systems are calculated. In the joints of the robot, a built-in tilt sensor is described in [47], [48]. The local environment map (shown in [49]) is updated using the data on items seen in the field of view of the cameras. The foot reference frame alters its position in relation to the surroundings when each step is taken since it is always centred on the foot that is presently in motion. The dead reckoning mechanism of the robot's computation is based on the data it provides, as described in detail in [50], [51]. The camera system must be positioned such that the most important elements of the walking route are visible in [52] due to the cameras' constrained field of vision. It is accomplished via the sophisticated view controlling (shown in [53], [54]) the angle of the pan and tilts in accordance with the orientation of vision that provides the greatest visual material for the current movement job.

**Figure 4:**
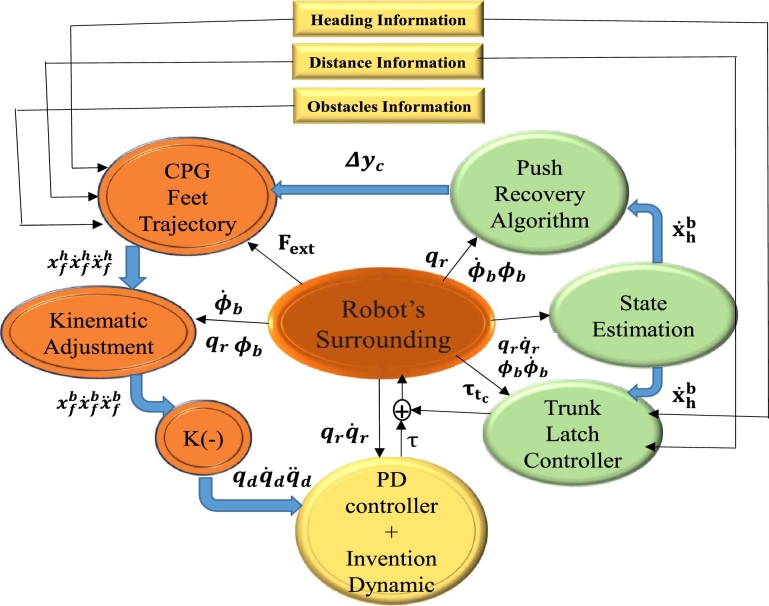
The Reactive Controller Framework (RCF) and the knowledge of the vision process are coupled. The motion production and motion control schematic get spatial information from the vision block, which is depicted in the head portion and golden colour.

The robot locomotion controller receives the set of instructions for the next step, which it then uses to create the proper paths used by the robotic joints [55], [56]. To reorient one's movement and maintain the machine on the intended route, details regarding the robot's attitude toward the walking area border are employed [57], [58]. Fig. 4 shows the framework for Reactive Controllers (RCF) and the vision process knowledge. The motion production and motion control blocks get spatial information from the vision block, which is depicted in the head portion and is golden in colour. The locomotion parameter may be modified separately, much like the Central Pattern Generator (CPG) algorithm.

**Figure 5:**
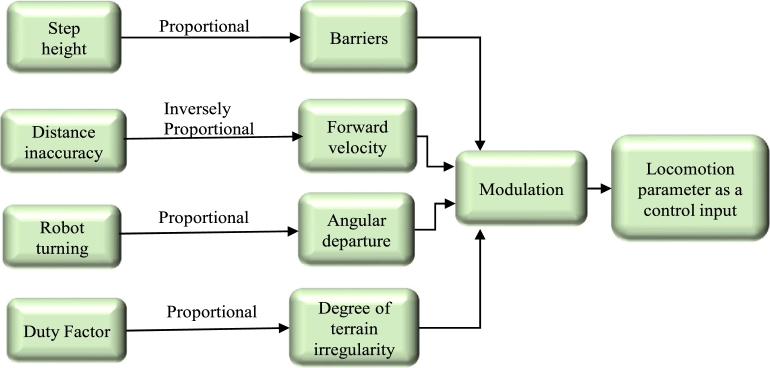
Control movements are produced using the locomotion measure as a control input and sensory data.

Using the visual data shown in Fig. 5 [59], we demonstrate the idea of considering each locomotion parameter as a control input and creating control actions to change them.

The CPG block receives information about terrain irregularities, the robot's relative position, and the direction of departure, as shown in Eq. (1) and Eq. (2), from a specific component of the locomotion creation and management algorithms [60].

(1) $$v_{d}=-k_{P}\theta_{h}$$

(2) $$v_{f}=kp_{v}\left(D_{d}-Df\right)$$

where $v_{d}$ and $v_{f}$ are the desired turning velocity and the desired forward velocity, respectively. The vision process provides the heading angle $\theta_{h}$ and the target distance Df. The parameters $k_{p}$, and $kp_{\text{v}}$ are controller gains. $D_{d}$ is the desired distance from the target. A proper step height is essential for lowering the possibility of foot-object frontal collisions and conserving energy during the leg swing phase, according to the RCF concept. Equations are used to represent both of the control laws indicated in Eq. (1) and (2). Following that, the trunk controller calculates joint torques to apply forces and moments based on $v_{f}$ and $v_{d}$ faults, i.e.,

$$F_{v_{f}}=k_{f}\left(v_{f}-{\overset{˙}{x}}_{b}^{h}\right)M_{v}=k_{v}\left(v_{d}-\overset{˙}{v}\right)$$

where the force and moment delivered to the trunk to minimise motion faults are denoted as $F_{v_{f}\;}$and $M_{v}$, respectively. The real robot rotation is shown by $v_{d}$, while the actual forward velocity is indicated by ${\overset{˙}{x}}_{b}^{h}$. Controller gains are the $K_{f}$ and $k_{m}$ parameters.

#### 3.1.1 Push recovery force and torque control

Dynamical balancing force control is crucial to regulating the motion of the robot's centre of mass and angular momentum [61]. Using a model technique for calculating the torque that is produced at all joints, it is possible to find the desirable centre of pressure and position of the next footstep [62].

**Figure 6:**
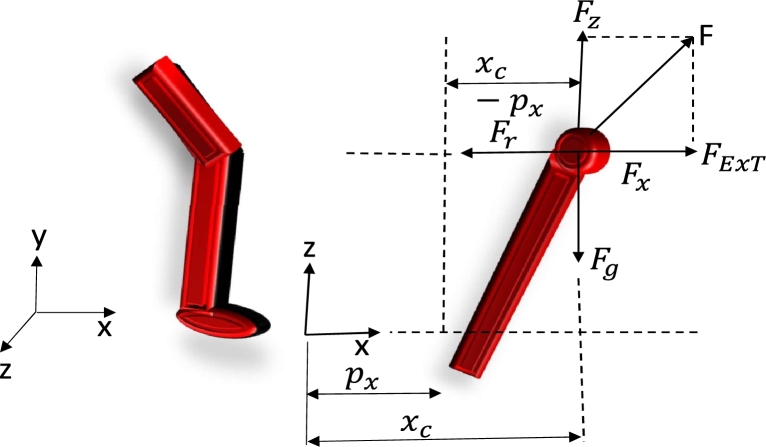
Robotic leg with push recovery and complete body mechanism of force control.

Fig. 6 shows the management of push recoveries for a legged, leg-controlled robot. The robot will collapse if the recovery controller is not pushed. A legged robot recognizes a push and an impending fall and selects a foot position to help it restore balance.

**Figure 7:**
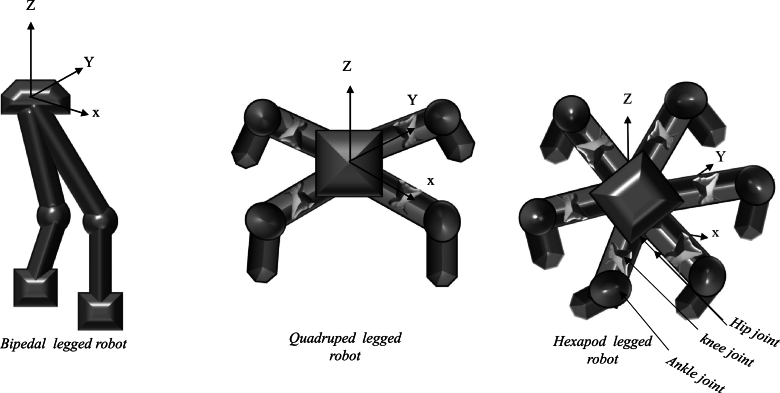
Motion mechanisms of various types of legged robots.

Fig. 7 shows a basic concept of a bipedal, quadruped, and hexapod robot. Using the robot's kinematic model, we can quantitatively evaluate the robot's speed, acceleration, attitude, and other characteristics. In order to develop the kinematic model of the hexapod robot, three coordinates are defined as the knee, ankle, and hip joint, correspondingly [63]. The reference coordinate is O. For each coordinate, the Z axis and the joint axis are parallel [46]. The X axis is the perpendicular that connects the axes of the jth joint with the (j + 1) joint. The Y axis is then calculated by using the right-hand rule.

### 3.2 Controller

The robot controller receives visual information from the tracking device's 3D coordinate item, its height, and the robot's travel length. These values are correctly translated from the camera reference frame into the robot base frame. The precision for the near-field, object level is around 2 cm.

**Figure 8:**
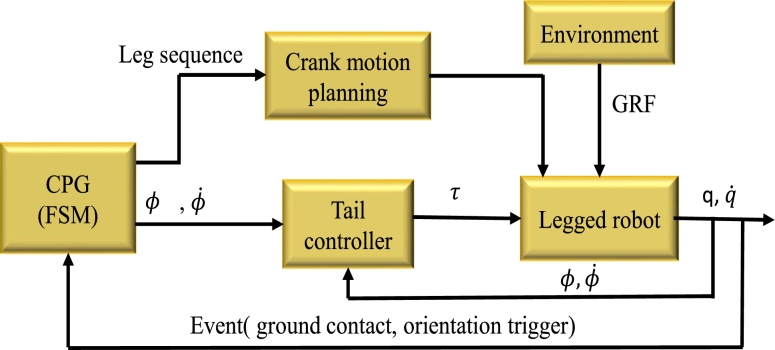
New legged robot controller architecture.

The inherent locomotion paradigm requires a controller, which is shown in Fig. 8, that synchronises the action in the tail and legs. A central pattern generator module schedules parameters for the legs and tail. The leg controller receives the timing data from the CPG component and transforms it into the crank's real direction. The control objectives are mainly the desired torso orientations $\phi_{d}$ and the angular velocity ${\overset{˙}{\phi }}_{d}$. The quadruped interacts with its surroundings and completes its movement tasks using information from its legs and tail.

### 3.3 High locomotion

A sequence diagram is the FSM (finite state machine), with movements among states acting as borders and limited variables acting as vertices. Transitions are frequently initiated by specific state-level events, such as the landing of a certain foot or the moment when the torso achieves a particular pitch angle. The walking steps of the bipedal and hexapod robots are shown below.

#### 3.3.1 One step jumping of bipedal robot

**Figure 9:**
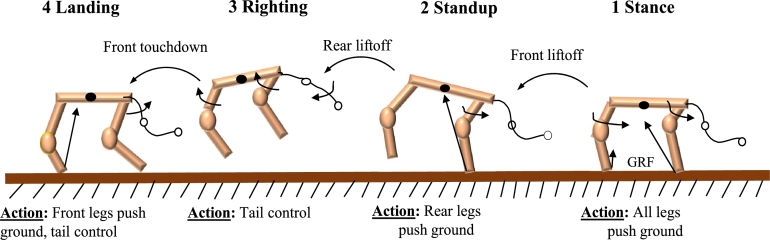
Action pattern representation for one step jumping.

Fig. 9 illustrates the four states of the FSM, which correlate to the four feet: stance, standup, righting, and landing. The same FSM seen in [64] might be used to produce several movement designs.

#### 3.3.2 Walking of hexapod robot during one cycle

The term “alternated tripod” refers to the gait seen in certain insects. It is evident from this gait that the legs in groups of three alternate between the transfer and support phases at each instant, which is depicted in [65].

**Figure 10:**
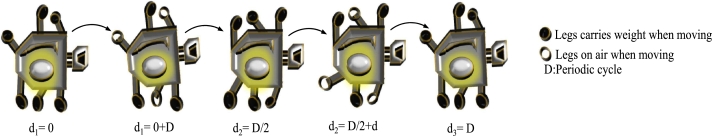
The configuration of the hexapod's walking step in a complete cycle.

The walking gait of the hexapod involves using three legs at once. As shown in Fig. 10, this results in a complete cycle with two phases: Legs 1, 2, and 3 support the platform during the first phase, during which it moves a distance denoted by *λ*. Between times $d_{1}$ and $d_{2}$, legs (4, 5, and 6) are raised off the ground throughout this period, following a cycloidal trajectory.

In the second phase, the roles are reversed and the cycle from phase “1” is repeated. Now that legs 1, 2, and 3 are elevated above the floor, legs 4, 5, and 6 assume the function of supporting legs. As Fig. 10 illustrates, phase “2” takes place in the space between times $d_{2}$ and $d_{3}$.

### 3.4 Control of tail

In robotic locomotion, tails are utilised for diverse purposes, such as providing normal force in climbing, hydrodynamic turning in aquatic robots, and controlling yaw turning through angular momentum exchange during airborne manoeuvres [66]. Therefore, the tail control method is of great importance for the next generation of legged robots. In order to move the torso into the correct orientation, the tail controller generates attempts to regulate particular tail joints [67]. The known methods (with pendulum tails) consist of movement path planning and momentum-guided nonlinear feedback control [68]. The torso orientation, which is one component of the entire state q, is the sole dynamic we are interested in since the methodology is based on partial feedback linearisation (PFL) [69].

The data will serve as advice for hardware design, nevertheless, as our objective is to examine the overall implications of various tail factors (like the tail's length). To derive the PFL controller, the system output is constructed as

$$y=q_{s}-q_{d}(t)$$

here $q_{s}$**=Sq** are the partial states to be linearised and indicates its dimension.$q_{d}$ is the reference trajectory and $s=\partial q_{s}/\partial q$ represents the selection matrix.

Then, because a spring-damper system is known to be exponentially stable, the output dynamics are built as such:

(3) $$\overset{¨}{y}+k_{d}\overset{˙}{y}+k_{P}y=0$$

Where $k_{d}=k_{d}I_{s\times s}$ and $k_{P}=k_{\text{P}}I_{s\times s}$ with $k_{d}$, $k_{P}$ **> 0**. Considering that the addition only exists in motion while the tail controller is activated, solving the equation For $\overset{¨}{q}$ and using $q_{s}=sq$ yields

(4) $${\overset{¨}{q}}_{s}=SH^{-1}(J_{\text{ta}}^{T}\tau_{\text{ta}}-C)$$

Substituting Eq. (4) into Eq. (3) and solving for $\tau_{\text{ta}}$**,** the tail controller is obtained as

(5) $$\tau_{\text{ta}}=X^{+}\left(SH^{-1}C+{\overset{¨}{q}}_{d}+k_{d}\left({\overset{˙}{q}}_{d}-{\overset{˙}{q}}_{s}\right)+k_{P}\left(q_{d}-q_{s}\right)\right)$$

In which **X=**$\;\text{S}H^{-1}J_{\text{ta}}^{T}$ and $X^{+}$ is the Moore-Penrose inverse of **X**.

The tail controller in Eq. (5), which was required to stabilise the bipedal walking standing there, is no longer required under these conditions. In these cases, the tail should just return to its original location. The tail controller thus functions as a purely damping system depicted in Eq. (6).

(6) $$\tau_{\text{ta}}=-\left[k_{d^{1}}\overset{˙}{\alpha }\;k_{d^{2}}{\overset{˙}{\beta }}_{1}\;k_{d^{3}}{\overset{˙}{\beta }}_{2}\right]$$

Where $k_{d^{1}}$, $k_{d^{2}}$, $K_{d^{3}}>0$ are the damping coefficients. Since pure damping consumes energy, the established stability will not be avoided.

## 4 Motion control

### 4.1 Gait generation

Gaits, which are different forms of mobility used by animals, are characterised by their movement styles. For terrestrial animals, the sequence of footfalls serves as the primary gait identifier. Gait is traditionally described using footfall patterns which are temporal. The precise left-right-left footfall sequence is seen in bipedal walking and running, for example, with the first contact phases happening at 0 and 50% of the stride duration.

**Figure 11:**
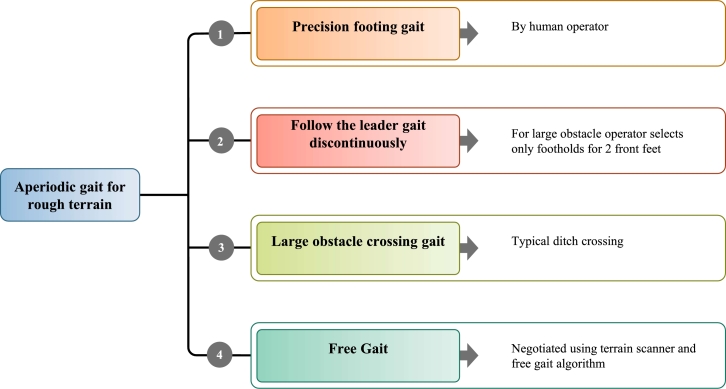
Gait control for rough terrain robot.

The classification of gaits as asymmetrical or symmetrical is usually based on the phase correlation of the left-right leg pairs. If one set of the left and right legs move in sync with each other by one-half stride cycles, the gait is said to be symmetrical. All conventionally symmetrical bipedal running, quadrupedal pacing, camel pacing, and hexapod trot of cockroaches happen due to the left-right pairs in the fore, mid, and hind legs being out of phase by one-half cycle from each other. No matter how many legs there are, gait symmetry is determined by the relationship between the left-right legs' half-cycle phases at a specific craniocaudal posture. In multilegged robots, gait refers to the specific coordination and sequence of leg movements during locomotion. The periodic and aperiodic gaits are the two main categories. Smooth and level terrain is best suited for periodic gaits, whereas rugged terrain is best suited for periodic gaits (e.g., rocks, soils, sands, slanted surfaces, and ditches). A robot's locomotion may require it to follow straight lines, climb and descend slopes, make turns, or travel at a specific crab angle. Only a few of the significant level-walking gaits on a variety of surfaces allow for such motions. The authors in [70], [71] introduce 3-DOF walking robot prototypes with vertical legs, rotary foot, and controlled mass, showcasing the capabilities for flat surface movements, direction changes, and rotation, suggesting potential for stair climbing. Dynamic gaits move quickly and have noticeable dynamic effects when accounting for inertial forces compared to static gaits' moderate motion and insignificant dynamic effects during locomotion. A challenging task is to produce dynamic gaits for a multi-legged robot moving across space with some slopes, ditches, and staircases. Fig. 11 demonstrates the gait control mechanisms designed for robots operating on rough terrain.”

### 4.2 Common patterns of gait

Animals walk or run with a specific gait on the ground. Running gaits include trotting, cantering, and galloping. Quadrupedal animals use symmetric and asymmetric gaits for their slow and swift running. The two most common gaits for running used by quadruped animals are trotting and galloping, used for moderate and high-speed running, respectively [72]. For instance, with a Froude number of 2-3, quadrupedal animals frequently go from trotting to galloping [73]. Despite variances in body structure or morphology. The whole-body mechanics of 2, 4, or 6-legged runner animals can be indistinguishable. This trait makes it possible to create and operate hexapod and quadrupedal robots utilizing similar technologies, such as the spring-loaded inverted pendulum (SLIP) model. Algorithms for single leg control may be employed to operate quadruped, and hexapod robots [74], [75] for gaits that operate the support legs one by one, like trot using diagonal pairs, lateral couples' pacing, and bound accompanied by forefront and rear pairs. In this paper, we considered the 20 most prominent legged robot models.

The dynamics of a particular gait can only be understood by measuring the forces acting on the Centre of Mass (CoM). This section explains how to measure CoM dynamics by combined leg force to identify oscillations. The RIP and SLIP models of gait are two standard models that are then discussed.

The simplest measurement of gait dynamics treats the centre of mass like a point mass and disregards any rotations about it (CoM). A rigid-body model must estimate the torques and forces independently on each leg in order to take rotations inside the CoM into account in addition to translations. When moving forward during leg locomotion, the Centre of Mass (CoM) oscillates vertically; if not, the CoM undergoes a net increase or fall throughout each step. The link across the relative phase, duty factor, and the dynamics of the centre of mass is explained by how the order and length of limb contacts affect the pattern of integrated forces acting on the centre of mass. Because each leg moves as one unit during bipedal hopping and quadrupedal galloping, the CoM oscillates vertically once every stride. Symmetrical gaits have two vertical oscillations, with one oscillation contributed by each leg. Mid-stance walking and running provide an increase in vertical acceleration in both uphill and downward motions. Walking and running have always been viewed as opposing one another based on traditional observations of the CoM's vertical position at mid-stance [76]. The three models of locomotion known as SLIP, RIP, and BSLIP are the most influential frameworks for studying gait dynamics, each addressing different aspects of locomotion. They are used depending on the kinetic and potential energy phase correlations between running and walking. The spring-loaded inverted pendulum (SLIP) model studies bipedal, quadrupedal, and multi-legged locomotion [61]. In midstance, both running and some other SLIP-like gaits achieve low kinetic and potential energy, putting these energies almost in sync. These spring-like dynamics offer potential energy savings if there are actual leg springs. The potential energy tends to peak around the middle of the stance, according to the theory of walking, throwing potential and kinetic energies somewhat out of phase [77]. Due to its twofold support and m-shaped force profile, the bipedal SLIP (BSLIP) model is more accurate than the rigid inverted pendulum (RIP) model.

### 4.3 Control of gait

For legged robots, there are many challenges in motion planning and control. These include whole-body encounters with alien environments as well as high-dimensional systems with redundant DOFs and a floating foundation. There have been several ways of controlling gait that fall into three categories: kinetostatics-based, dynamics-based, and model-free methods [101]. Static gaits often employ kinetostatics-based control algorithms to project the centre of gravity (COG) and determine the zero-moment location (ZMP). Galloping and trotting gaits are not suited for kinetostatics-based approaches. Because legged robots move quickly, the ZMP computation lacks an explicit polygon. Because the robot's torso must be accelerated and slowed right down in each gait step, the ZMP control approach is inefficient.

The stability criterion is a key point of comparison between static and dynamic gaits. Continually moving feet or torso allows a legged robot to maintain dynamic stability for dynamic gaits. In comparison to kinetostatics-based control, the two dynamic-based methods are more effective for legged robot high-speed control. Yet, there is still disagreement about the dynamic stability requirements for legged robots. In this part, dynamics-based models will be examined, including the simple SLIP control model, as well as more complex models like model predictive control (MPC), whole-body control (WBC), and virtual model control (VMC). Also included are two well-known model-free approaches (RL): central pattern generator control (CPG) and sim-to-real reinforcement learning. This paper considers the following legged robots Quadruped, Minitaur, SCOUT II KOLT, Hexbot-IV, HyQ, Cheetah 1, Cheetah 2, Cheetah 3, StarlETH, and ANYmal to give an overview of their gaits.

SLIP model was suggested by Cavagna et al. [76] to imitate the dynamic traits of many animals during locomotion. When running, they exhibit diverse patterns of force and motion. Walking causes the torso's potential and kinetic energy to shift either sinusoidally or out of phase. A point mass designs the passive and conservative SLIP model on top of a spring. The body moves on a ballistic trajectory under the influence of gravity, and the springy leg adjusts its landing angle to leap to the appropriate position during the fight phase. Three servo loops are predetermined by Raibert et al. [26], [74], to regulate hopping height, body attitude, and running speed.

Virtual model control (VMC) is a simple method of controlling movement on two legs. A virtual model is used to generate the actuator torques in between contact points of the spring, mass, dashpot, damper, non-linear potential, latch, bearing, and dissipative field. The development of mappings across virtual torso forces and torques of virtual leg forces, joint torques [90], and a whole-body VMC strategy for four-legged robot trotting with a focus on rotation upon the body's diagonal line are recent improvements in this technique [102].

Model predictive control (MPC) is a technique for iteratively resolving mode-based optimization issues by taking into consideration the current state of the system and projecting how it will change in the future. MPC has been widely utilized to formulate and optimize ZMP footsteps in humanoids [102], but it is rarely used for multi-legged robot gait planning [81]. However, with appropriate linear constraints, it can be used to create stable motion by optimising states with control inputs for a finite horizon [103]. Recently, it has been possible to lower the computational cost of online MPC techniques by utilising interior point and active-set solvers. Open-source problem solvers notably qpOASES and ECOS are capable of providing quick and trustworthy MPC issue solutions [104], [105]. Carlo et al. [86]found that a precise simulation of the dynamics of a legged robot over the span of the prediction horizon is not as crucial as an accurate simulation of its dynamics in the instance. As a result, one may refer to the dynamic control of a multi-legged autonomous robotic system based on the MPC technique as a convex optimization. Convex MPC can be used to manage ground response forces and produces very robust dynamic locomotion at various speeds.

Whole-body control (WBC) is a control architecture providing precedence amongst tasks for complicated tasks [106], [107]. Since motion planning and motion control are separated, it is simple to complete several jobs while still taking into account the characteristics of the legged robot. A task space decomposition technique was suggested by Farshidian et al. [108] to break the connection involving contact force and non-contact control systems. The intended motion tasks for all joints may therefore be accomplished using WBC's formulation of locomotion control like an optimization problem that takes into account the entire dynamics of the legged robot. Due to the computing demands, solving the optimization issue in real-time is difficult. Using convex cost functions such as quadratic programming (QP) and a control loop in real-time operating at the 1 kHz level [109], [110], the WBC and hierarchical optimization (HO) could be applied to a legged robot to achieve excellent locomotion capabilities, enabling them to navigate invisible obstacles without the use of motion planning. Table 3 shows these dynamics-based control methods of some existing legged robots.

**Table 3:** Dynamics-based control methods of some existing legged robots.

	Reference	No. of legs	DOFs of leg	Gaits	Control methods
Cheetah 1	[78], [79], [80]	4	2	Gallop	Proprioceptive impedance control

Cheetah 2	[81]	4	2	Jump over obstacles	MPC

Cheetah 2	[82], [83], [81]	4	2	Bound	Direct ground reaction force control

Cheetah 3	[84], [85], [86]	4	3	Trot	MPC
				Flying-trot	MPC
				Gallop	MPC

ANYmal	[87], [88]	4	3	Trot	WBC

StarlETH	[89], [90], [91], [92]	4	3	Trot	WBC

Quadruped	[74]	4		Trot	SLIP

SCOUT II	[93], [94]	4	2	Bound	SLIP

Minitaur	[95], [96]	4	8	Pronk	SLIP

Hexbot-IV	[67]	6	3	Trot	SLIP for hexapod tripod gait

HyQ	[97]	4	3	Trot	VMC

HyQ	[98], [99]	4	3	Trot	Active impedance control

KOLT	[100]	4	3	Trot	SLIP + Fuzzy Control

In multi-legged robots, model-based techniques have produced excellent control performance. However, they have two key drawbacks: limited precision and labour-intensive development. The legged robot being controlled using model-free control techniques has neither a kinematic nor a dynamic model. Modern optimal controllers now include learning models, making them a desirable technique for controlling dynamic locomotion. To brief the model-free control methods, we analysed the following models: Cheetah-cub, Tekken 1, Tekken 2, HyQ, Baby elephant, Minitaur, and ANYmal.

Central pattern generators (CPGs) are made up of oscillators in the spinal cord and neuron pools. They produce rhythmic control signals to manipulate the flexor muscles and the leg extensor [118]. CPG and reflexes work together to largely produce animal walking [114]. The term “reflex” in robot control refers to the joint torque production that results from sensor data and the reaction as CPG phase modification depending on sensory feedback. Using just descending control signals, CPG controllers are able to execute well-coordinated leg motions and gait changes. External sensory data, such as leg loading, touchdown feedback [114], [116], [119], and robot torso postures, paired with another control technique [113], [117], [120], [121], [122], [123], are two recent tendencies for CPG control. By using the connected NOs' limit cycle behaviour to provide joint control in real time, CPGs offer an innovative method for generating gait trajectories.

Sim-to-real reinforcement learning (RL), a data-driven technique, claims to get over the drawbacks of model-based methods [124], [125], [126]. The “reality gap” between actual and simulated systems, however, is a result of things like improper model parameters, overlooked dynamics, and calculation mistakes. RL algorithms have been created to deal with autonomous locomotion issues in order to solve this. Inertial parameter identification by Lee and Park [127] increased the model performance of a high-DOF-legged robot. Tan et al. led the method of introducing perturbations in a small observation area and randomisation of the physical settings to the creation of a resilient controller [128]. A successful learning strategy to manage a high-performance locomotion controller using SEA actuators was recently proposed by Hwangbo et al. [111]Identification of the robot's physical characteristics, estimation of its uncertainties, and training of an actuator net featuring intricate actuator dynamics. The physical system was used to train and implement the control policy.

**Figure 12:**
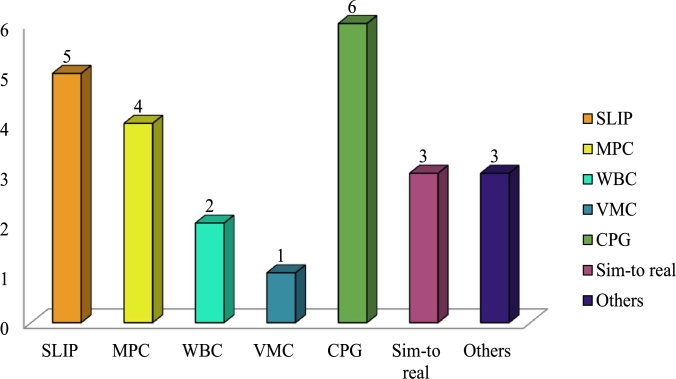
Control method using frequency for existing robots.

Reinforcement learning (RL) from simulation to reality has advanced significantly in recent years. RL frequently requires several weeks or even months of learning before it can produce extremely flexible and effective algorithms. There exists a “reality gap” between real-world and simulated systems as a result of problems including improper model parameters and imprecise dynamics, including calculation errors [101]. Sometimes enhancing simulation quality or the controller's resilience to oscillations can erase the reality gap. Table 4 shows the use of model-free control methods for some existing legged robots. Fig. 12 shows the frequency of use of all the above-mentioned legged robot locomotion control techniques among the legged robots mentioned in Table 3, Table 4. And Table 5 discusses their advantages, limitations as well as applications.

**Table 4:** Model-free control methods of some existing legged robots.

	Reference	No. of legs	DOFs of leg	Gaits	Control methods
ANYmal	[111]	4	6	Flying-trot	Sim-to-real R

Cheetah-cub	[112]	4	8	Trot	CPG

HyQ	[113]	4	12	Trot (On unexpected territory)	CPG-Task space trajectory generation

Tekken 1	[112], [114], [115]	4		Trot	CPG
				Bound	CPG

Tekken 2	[112], [116]	4	4	Trot	CPG

Minitaur	[14]	4	8	Trot	Sim-to-real RL
				Gallop	Sim-to-real RL

Baby elephant	[117]	4	6	Trot	CPG

**Table 5:** Gait control methods and their advantages, limitations and applications.

Gait Control Methods		Advantages	Limitations	Applications
Kinetostatics-based methods		1. Robotic quadruped and hexapod walking at moderate speeds under control [109][129]	1. Not suitable for running gaits 2. Not energy efficient 3. Legged robot's heavy torso needs acceleration and deceleration in every gait stride	1. Zero moment point (ZMP) detection 2. Provision of center of gravity (COG) projection with static gaits

Dynamics-based methods	SLIP	1. Simple 2. Robust dynamic gait with high performance 3. Tolerance for small disturbances	1. Control precision in tracking the desired velocity 2. Highly dependent on tuning or optimization	1. Simple dynamic gait control
	VMC	1. Easily describes complex tasks 2. Computations of a relatively small amount 3. Ability to perform complex control tasks	1. Complex computational processing 2. Limited adaptability to unfamiliar surroundings [130]	1. Actuator torque computation for static gait control 2. Locomotion balance control, steering and so on [131]
	MPC	1. Generate stable motions 2. Optimally manage states and regulate inputs for a finite horizon 3. Rapid, reliable and decreased computational expense of online MPC	1. Problems in models and constraints have to be solved online 2. Applicable only in environments with high performance processing hardware 3. Complex and time- consuming computation [66]	1. Route tracking of mobile device
	WBC	1. More capable of dealing with almost all constraints, 2. Increases compliance and decreases motion planning complexity 3. Upper-level control execution with abstract planning development	1. Generating control signals for all moving joints are difficult to implement	1. Complex movement activities with task prioritization

Model-free methods	CPG	1. Well-coordinated moves with physical communication throughout the body 2. Gait changes with straightforward descending control signals 3. Use of reinforcement learning (RL) neural oscillator (NO) to acquire and enhance various parameters online 4. Lesser dimensional control while maintaining highly flexible gait patterns	1. Declination in legged robot's compliance with feedback and position control	1. Recurring tasks 2. Adjustment to unstructured environments
	Sim-to-real RL	1. Coping with autonomous locomotion problems via data driven algorithms 2. Avoiding limitations of model based approaches	1. RL typically requires long training time to generate highly agile and efficient algorithms [132].	1. Autonomous locomotion issues [125]

## 5 Evolution of legged robots

Engineers, as well as scientists, have studied the development of robotics, noting advancements based on robot generations. Kato and his group at Waseda University presented the first humanoid robot in Japan, WABOT 1, in 1973 (shown in [133]). The very first active exoskeletons and several other devices, including the Belgrade hands, were created in Serbia by the Mihailo Puppin Institute [134], [135]. This was the first endeavour to formally state that legged robots need dynamic stability. The first computer-controlled walking device was developed thanks to the groundbreaking work of M. Raibert and R. McGhee [136]. Notable accomplishments were made in the 1960s and 1970s at Carnegie Mellon University in Pittsburgh and USC, including a two-legged hopping robot, a well-known flip and a series of robots with one, two, or four legs designed in the 1990s. The early 1990s marked a third crucial era for legged robot research. Following in McGeer's footsteps, several researchers have made numerous extensions, such as adding the trunk, knees, or feet, which were used by Rabbit (2003) to use semi-passive control to walk and run underactuated robots. Honda created P2, the first humanoid robot in Japan, in 1996. The most astounding technological advancements are still being made today by industrial enterprises like iRobot, NVIDIA, Boston Dynamics, and so on. The concept of robot generations began with first-generation robots being basic mechanical arms. Second-generation robots may communicate with one another without being continually monitored by a human operator. There are two critical paths for developing smart robot technology of the third generation: autonomous robots and insect robots. A fourth-generation robot is any robot that has not yet been substantially implemented.

### 5.1 Generations of legged robots

* Robots of First Generation
  A moving robot with 1,000 MIPS (millions of instructions per second) computer capability can keep a rough map of its surroundings. When not engaged in its specific robotic activities, the robot should be able to connect to personal computers via wireless networks. Its mobility should be effective on level ground, where most chores occur. It should also be dependable and safe on stairs and rocky terrain. More important information about the first-generation leg robot is described in this paper [137].
* Robots of Second Generation
  The goal of adaptive learning is to assess the impact of each action in a given situation. If a piece of software is constantly producing unfavourable outcomes, the conditioning system may eventually suppress them. The numerous independent programs of a condition suite, each reacting to a different stimulus, interact with one another as well as with the robot's monitoring programs and surroundings, as described in detail in [138].
* Robots of Third Generation
  A robot with a simulation on board will keep a running record of what is happening around it. As each item is recognized and associated with its appropriate interaction and perception primitives, this will enable a robot's three-dimensional mapping of a room to be converted into a functional model. It will be as powerful as a third-generation universal robot, as opposed to the current supercomputers, which optimize second-generation applications. These papers [139], [140] contain more detailed information about the third-generation leg robots.
* Robots of Fourth Generation
  Robots will have processors strong enough to replicate the environment while reasoning about it. Fourth-generation robots will be able to create ultra-sophisticated robot programs for other robots or themselves, as shown in [141]. Simulator-augmented language comprehension and reasoning in robots may be so successful that they will be accepted for use in the standard computer programs [142]. A fourth-generation robot will resemble us in some aspects while being unlike anything the world has seen before.

### 5.2 Add-on components for next generation legged robots

**Figure 13:**
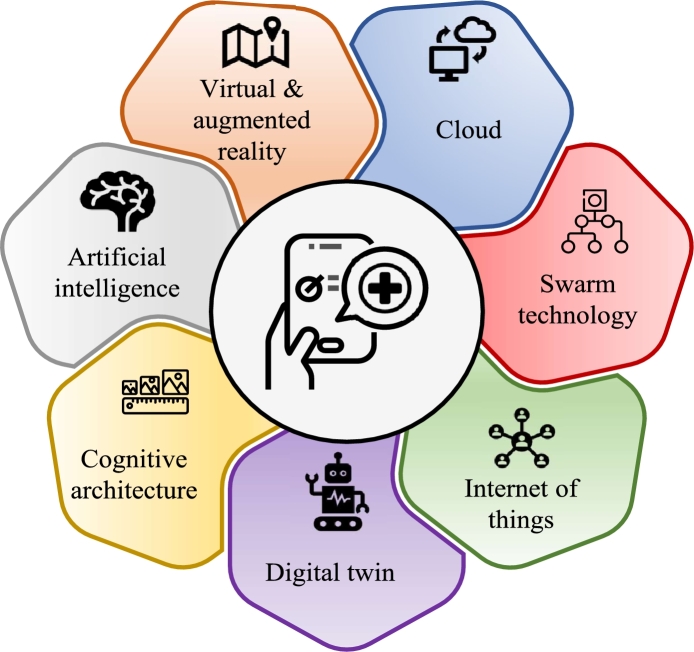
Add-on components of next-generation of legged robots.

Developments in related technology have affected the functionality of future-legged robots. In the upcoming years, mobile robotics and robotics, in general, will continue to develop [138]. The next generation of legged robots must have the same elements that make up the current ones. Nevertheless, they will incorporate a few additional technologies as improvements as demonstrated in Fig. 13.

* Cognitive Architecture and Artificial Intelligence (AI)
  The cognitive and AI components are more closely tied to mobility when considering legged robot developments for various applications. Because they possess the internal ability to recognize obstacles and decide how to react to them, collaborative mobile robots that have found their way into contemporary fulfilment centres are able to explore any unknown terrain autonomously. Such a significant improvement in mobility makes it possible for mobile collaborative robots to enhance the fulfilment process. AI also enhances material use and energy management in mobile robots. It refines route planning, terrain analysis, and energy conservation. Shared resources improve efficiency, and machine learning predicts energy use accurately [143].
* Internet of Things (IoT) and Cloud
  IoT provides a mechanism to communicate with and learn more about a process in real-time. It has made it possible for a new kind of robot to evolve that is better suited to work with humans than to function independently in a setting devoid of them. IoT enables data offloading to stationary computers, reducing onboard processing and enhancing efficiency [143]. Furthermore, whenever there is an internet connection, enterprises can monitor, operate, and manage robots thanks to the cloud. This may make workplace robots more helpful and assist robot producers in providing better customer service. For instance, manufacturers may utilize the Cloud to take control of a mobile robot that has encountered a problem without disturbing the consumer and remotely check on the health of robots owned by their customers.
* Digital Twins
  The digital twin (DT) method builds a perfect digital replica of a legged robot using real-time data from actual Internet of Things (IoT) sensors. It may be used to imitate the actual IoT device and offer in-depth information on live applications, performance, and potential problems. In a virtual, digital, or cyber world, digital twins can help in finding and contrasting various use cases, identifying and testing new settings, establishing diverse scenarios, and spotting novel challenges. They can also serve as a platform for human-robot communication and a genuine, practical robotic tool that advances the development of robotic legs in the future.
* Virtual and Augmented Reality
  The integration of immersive technologies (such as VR and AR) into human-robotic device interfaces and robotic device interactions is made possible by increased cognitive capacities at the edge of the next generation of legged robots. Applications for legged robotics can integrate VR/AR for learning, navigation, and support features. While AR overlays computer-generated information on the actual world, VR replicates the surroundings. In order to train and validate algorithms for perception, motion planning, and control, the AR framework creates a representation of the physical environment. For instance, in a real-world outdoor setting, AR may assess a robot's capacity to plan a safe route to a target place while virtual things dynamically enhance the planning environment.
* Swarm Technologies
  The focus of swarm technologies and swarm robotics [144] is on investigating how intelligent systems made up of several autonomous robots are applied to carry out group activities. The fusion of swarm robotics technologies with multi-legged robots would enable them to have self-organising qualities for multi-robot systems with high redundancy and a need for scalability, adaptability, and resilience. The algorithms for flocking, dispersing, aggregating, foraging, and following trails are addressed by swarm technologies, which apply the dynamics of natural ecosystems that can lead to the creation of multilegged robot teams that exhibit emergent cooperation as a result of acting on preset interests and goals. Fig. 13 gives an overview of the key components and technologies driving the advancement of next-generation legged robots.

Decreasing the complexity of the design will be necessary for legged technologies to advance. For more than a century, engineers have worked to create machines with mobile legs, first focusing on linkage-based designs. However, researchers began looking at active control techniques, and gradually adaptive-legged robots started to appear. Legged robots must be able to handle large force and loading patterns having high bandwidth to allow quick replacement in flight while simultaneously weighing as little as possible. To overcome these problems, a newly developed technology called proprioceptive actuators is needed for better future work. It outlines the main problems with the legged robot actuator design and provides an alternative approach to reducing mechanical resistance and enabling dynamic physical contact via transparent force.

## 6 Critical requisites for next generation legged robots

**Figure 14:**
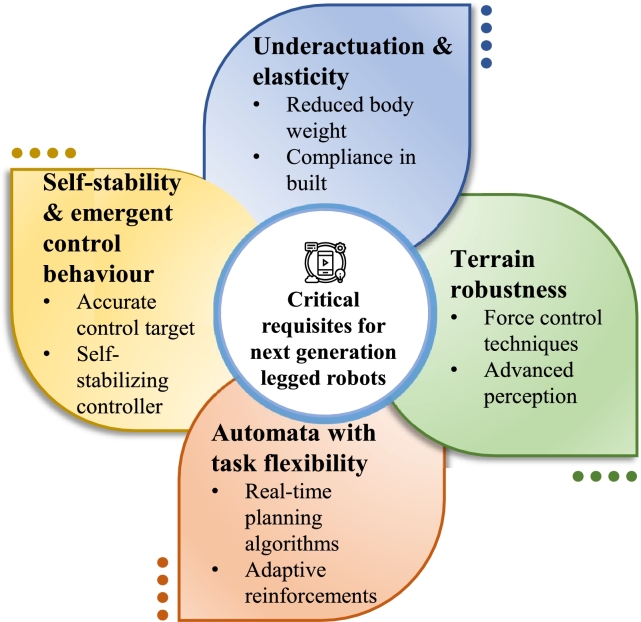
An Overview of the Main Applications and Challenges of Legged Robots.

The engineering of a robot with legs is complex, but the results have broad applications. It could be advantageous to venture into advanced-legged robotics. There are four requirements that will be crucial to the developments in the locomotion of next-generation robots. Fig. 14 provides a summary of these requirements.

* Underactuation and Elasticity
  Bipedal humanoid robots frequently require more energy in terms of magnitude to run and walk than humans of the same size [145]. Underactuation is an efficiency-driven strategy that entails developing bipedal robots with fewer motors. The Cornell Ranger, a primary underactuated walker, was able to set a distance record of walking 40 kilometres on a single battery charge without human interference [146].
* Terrain Robustness
  Real-world conditions require the use of robots that are resistant to unfamiliar terrain. Researchers have developed force-control approaches to locomotion. This enables legged robots to navigate various terrains without falling. The technique is also a helpful tool for delicately manipulating robotic arms in the context of manufacturing. A flexible design that allows for various modes of movement can also help in achieving terrain robustness. LEO, a versatile bipedal robot, autonomously combines walking and flight modes autonomously. Its unique design facilitates tasks on various surfaces, including rope walking and skateboarding, challenging for other bipedal robots [147].
* Self-Stability and Emergent Control Behaviour
  A bipedal robot named ATRIAS was programmed with a self-stabilising walking controller. It can methodically cycle its feet and manage its pace when advancing without the need for complex computing. When instructed to accelerate using this primary controller, it started running without receiving the specific command to do so [72]. This emergent behaviour may occur in a factory with the help of next-generation legged robots.
* Automata with Task-Flexibility
  Developing a task-flexible framework for controlling legged robots has become a vital step towards practicality. The demand for task flexibility has resulted in the growth of real-time optimisation approaches for generating stable controllers for a specific job. And so, optimisation-based techniques were the preferred options for the robotics challenge of the next generation. Fig. 14 shows the key critical requirements for legged robots of the future.

## 7 Next generation legged robots: challenges, roadblocks and applications

### 7.1 Primary challenges

**Figure 15:**
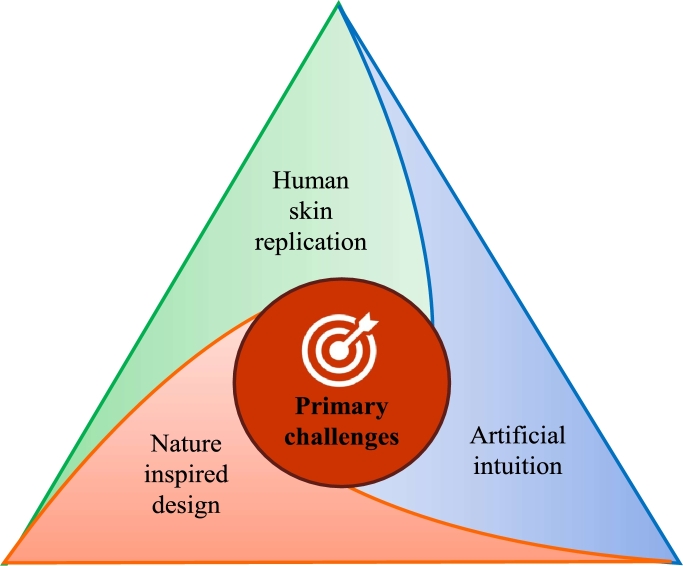
Primary challenges of next-generation legged robots.

The development of algorithms that allow the next generation of legged robots to navigate through complex terrains, including rough and cramped spaces, and operate in a semiautonomous manner, is one of the challenges they must overcome. Their functionality, as well as their capacity to perceive, plan, and effectively control their movements, depend on the achievement of high levels of stability and accuracy. It is also a significant challenge to develop the artificial intelligence and sophisticated sensors needed to support these robots' context awareness and successful navigation. Three basic challenges as shown in Fig. 15 for the design and development of the next generation of legged robots are discussed below:

* Human Skin Replication
  Legged robots with powerful optical and audio systems have been created thanks to recent advances in robotics. However, giving robots the ability to sense touch and human activity is still a difficult task. Safety, increased utility, and flexibility all depend on touch. Legged robots can be taught to pick up simple objects like cubes for recognition tasks, but it is difficult to pick up and recognize a complex structure or movement in unpredictably changing outdoor surroundings. The next generation of legged robots is anticipated to use touch to navigate a variety of items in uncharted terrain. The functionality of current primitive technology like force and pressure sensors is constrained. In order to give robots collaborating closely with people access to human skin, artificial tactile skin is necessary [148]. This demonstrates how crucial it is to create and apply such technology in the field of robotics. Robotic skin needs to meet a number of technological and practical requirements, including having flexible and elastic core sensors that can handle delicate and difficult objects. In order to protect individuals around them, robotic skins should also be able to tolerate prolonged mechanical stress and have 360-degree anti-collision sensors. This calls for the creation of artificial intuition and context awareness, both of which are essential for the next generation of legged robots. Although such technology is not yet available, it will be necessary for future advancements. It is a difficult problem to coexist and interact with people in the same physical environment without providing a possible hazard, but it is a necessary condition for the development of the next generation of legged robots.
* Artificial Intuition
  To enable good navigation in a variety of terrains, the next generation of legged robots will require a comparable sense of intuition and context awareness. To achieve previously unheard-of levels of engagement with their surroundings, the underlying artificial intelligence (AI) must be pushed to its breaking point. It is required to develop extreme-edge AI, which combines sensor input to make quick decisions without overwhelming the robot's mainframe. This means removing the need for robots to exchange and store a large number of unnecessary inputs, allowing them to live on batteries. In order to provide robots with artificial intuition, their AI capabilities must be crucial.
* Nature-inspired Designs with a Human Touch
  Theoretically, creating synthetic skin or giving artificial intelligence to robots might be very helpful. Yet, there are significant practical difficulties with putting these advances into practice. In this regard, nature and the sum total of human experience throughout history may provide insightful perspectives on these problems. In particular, the exceptional effectiveness of the human brain and body could be used as inspiration for extremely effective and energy-efficient robotic design. The development of robotic technology may have a big impact on how we serve those in need, help people in their daily lives, and keep people safe. We can envision a future where humans can actually thrive and work to build a more inclusive and equitable society where people can care by developing robots that are increasingly independent, flexible, and tactile.
* Learning and Adaptation
  A comprehensive strategy integrating modern technology and creative design approaches is needed to produce multilegged robots. This involves implementing continuous learning algorithms that enable robots to adapt and improve their locomotion performance through experience. Through feedback loops, the robot may modify its behaviour in response to past encounters with its surroundings. To overcome these obstacles, cooperation between specialists in robotics, control systems, artificial intelligence, and materials science is essential. Reinforcement learning and other advanced control algorithms improve coordination and adaptation to different terrains. Real-time environmental awareness is made possible by the integration of sophisticated sensors, including touch and LiDAR sensors. Resilience in dynamic contexts is ensured by fault-tolerant structures and redundant systems.

### 7.2 Adoption roadblocks

Although legged robots have great potential, there are still significant barriers that prevent their widespread use in a variety of sectors, such as manufacturing, healthcare, and military applications. The legged robots of the future must be able to lift heavy objects and adapt to the changing environment. Exteroceptive sensing provides a 3D map, and it is essential to plan, control, perceive, and assess the state. However, inaccurate maps, delayed controls, unforeseen occurrences, model flaws, and structure compliance can all result in errors in robotic systems. Moreover, the systems need to respond to human inputs quickly, adapt, and ignore disruptions. The functionality of legged robots is being worked on through ongoing research, but there are still many challenges to be solved. Such as:

* Employment and Human Contact
  Some early concerns about next-generation robots were centred on the human labour they replace and the loss of human engagement it may create. Careful supervision will be required during the rollout and installation of this technology, which must be tailored to specific locations to minimize short-term repercussions. In the long term, the arrival of next-generation technology will bring with it a slew of new sectors and employment growth, which, if well managed, can offset most of these effects.
* Moral Considerations
  There are several ethical problems when putting robots into contexts where they may make choices that impact human lives, such as the deployment of autonomous robots within the military or robotic surgeons. Legislation to handle these challenges will be required and this is expected to be a topic of intense discussion at the regional and international levels in the coming years.
* The General Public's Perceptions
  Education will be required to increase public knowledge and acceptance of robots in settings such as schools and hospitals and to explain both the potential and drawbacks of robot platforms.
* Security
  Cyber security is becoming increasingly vital as more autonomous robots work in sensitive and high-risk areas. There is a chance that someone may seize control of the robot and cause significant damage, weakening public opinion of robotics as a good technology.

**Figure 16:**
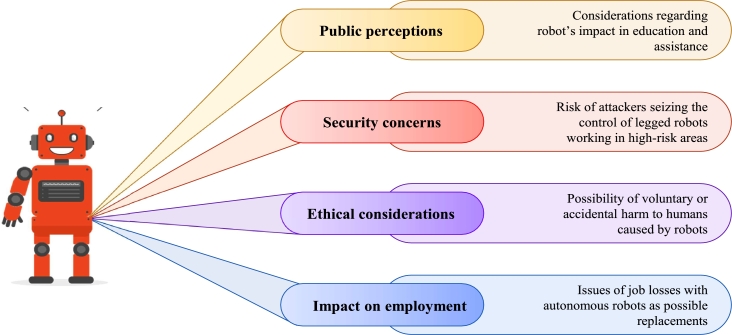
Roadblocks in the adoption of next-generation legged robots.

Next-generation legged robots face all these significant obstacles illustrated in Fig. 16 that need to be overcome before they can be fully integrated into a variety of industries, such as the high cost of production and maintenance as well as the scarcity of skilled workers.

### 7.3 Highly potential applications

**Figure 17:**
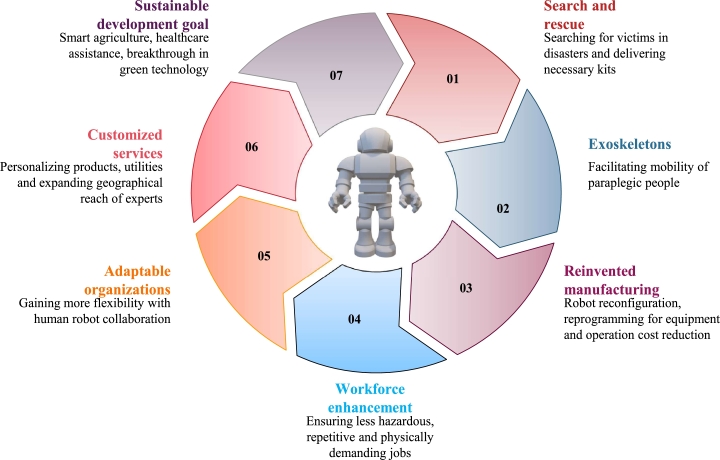
Potential applications of next-generation legged robots.

Robotics has already transformed and will continue to impact various industries, allowing robots to collaborate with humans in the workforce. The integration of robots into the workforce as collaborators alongside humans has the potential to impact all industries and is predicted to occur in both developed and emerging nations. As illustrated in Fig. 17 and from the given description below, autonomous-legged robots will be utilized in various ways in future:

* Robots in Hospitals
  One of the most promising applications for next-generation legged robots is in the healthcare sector, where they are making significant strides. Robotic surgery and autonomous mobile robots that can transport medical supplies and equipment inside of hospitals are just two examples of the notable increase in the use of robots in the healthcare sector. Beyond these crucial roles, robots are also taking on more routine jobs like guiding patients and guests, making it easier to navigate through large hospital complexes, and freeing up valuable time for highly qualified medical staff [149].
* Exoskeletons
  People who are paralysed from the waist down can now walk thanks to recent developments in exoskeleton technology, particularly the Phoenix exoskeleton [150]. We anticipate that exoskeletons will be used more frequently to aid in mobility and work-related activities as the price and weight of such devices drop.
* Reinvented Manufacturing
  In order to work alongside people in manufacturing lines, Rethink Robotics has developed the Baxter robot. In a manner akin to how young children are taught, Baxter is made to be reprogrammed by humans by having them physically move their arms into desired positions [151]. These robots may reduce equipment costs for manufacturers by facilitating human-robot collaboration, minimising the modifications required to buy and deploy the robot, as well as the costs associated with learning to operate and reconfigure it.
* More Customised Products or Services
  Robots collaborating with humans will make it simpler to customize and modify goods and interactions with specific customers and expand experts' geographical reach.
* Closed-loop Systems
  Robots may reduce the amount of waste in the production-to-consumption chain by improving manufacturing procedures and control and recovering and reusing materials and components at the end of their lives.
* Flexible and Adaptable Organization
  The mix of robots and humans may provide more flexibility than pure automation. Robot product generations may be remotely and instantaneously updated rather than having to be repaired or returned to the manufacturer.

The use of exoskeleton robots is anticipated to enable individuals to perform physical tasks more efficiently, while the implementation of artificial intelligence will aid in cognitive and decision-making tasks. This collaboration between humans and robots will result in new service delivery methods, personalized products, and solutions for unmet customer needs. Additionally, companies may experience increased efficiency, flexibility, and decreased barriers to entry. The impact of robotics is expected to extend beyond traditional industries, offering opportunities for start-ups and new tools for individuals in the workplace. Moreover, wheeled robots can only move in a restricted number of places, whereas legged robots are primarily intended for movement in unstructured situations. Legged robots are used in a variety of industries, such as mining, search and rescue, inspection, surveillance, and nuclear decommissioning, presenting significant benefits in challenging environments. Additionally, they can also help prevent workplace accidents by assisting human workers in hazardous situations as well as providing support in specialized settings like elderly care. Ultimately, the use of next-generation legged robots may enable the creation of novel business models. Hence, the control techniques of legged robot locomotion must be improved.

## 8 Road to next-generation legged robots

**Figure 18:**
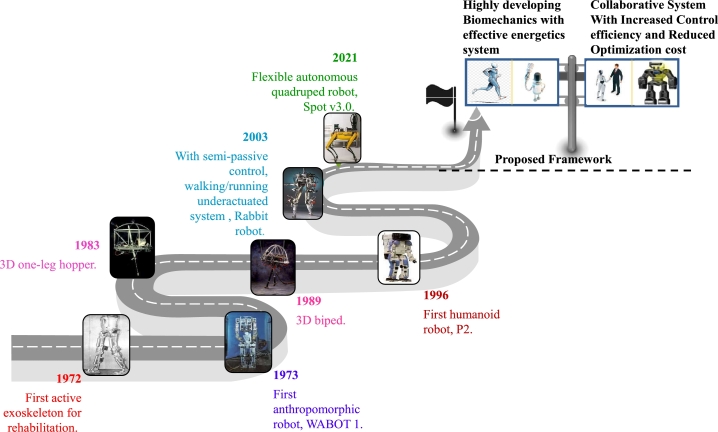
Road map to next-generation of legged robots.

Over time, substantial improvements and discoveries in the field of robotics have helped to accomplish a number of technological, theoretical, and industrial successes. Legged robots are among the most intriguing modern robotic systems due to their potential for autonomous navigation in difficult and constrained situations, such as uneven and rugged terrains. Fig. 18 illustrates the planned structure for the upcoming generation of legged robots and the development of this technology over time. Although a far-off fantasy in the past, inventive research's success has made it a reality. Legged robots give users the chance to explore previously inaccessible spaces and learn the fundamentals of safe and effective mobility. Current computational control and stability analysis tools, however, are severely hampered by their complex dynamics. Significant mathematical ramifications result from the requirement for sporadic touch between a walking system and its surroundings. The movements of legged systems have been observed to be remarkably precise and reliable in recent years.

However, the next generation of legged robots will need to overcome several significant obstacles, such as creating more effective and dependable actuators, creating sophisticated control systems that allow for more fluid and natural movements, and advancing sensor technology to give robots better situational awareness. Interdisciplinary cooperation between researchers in robotics, biomechanics, materials science, and other fields will be necessary to accomplish these goals. A wider range of researchers will be able to contribute to the development of legged robots and progress in this field will be accelerated by the creation of more accessible platforms and tools. Some crucial features that can help in identifying the differences between past, present and future generations are listed below to provide a requisite characterisation of the next generation of legged robots.

* Biomechanics
  In the past, robotic legs were mainly built with stick-like structures with some linear springs for a simple control scheme to actualize some slow steps while in static equilibrium. Then with the arrival of the zero-moment point (ZMP) concept, dynamic stability was formalized and later came active hopping-legged robots. Afterwards, numerous studies of purely passive mechanical systems for orbital stability have added extensions like trunk, knees and feet to underactuated walking and running robots. For example, in bipedal robots, the inclusion of knees for a human-like appearance often results in crouched walking. This design choice increases torque requirements and adds complexity, which can limit the robot's speed. Engineers and researchers are now looking at the biomechanics of various animals that are adept at navigating through rough ground and program the control algorithms gathering information from those biological sources for more effective locomotion, such as various animals that are skilled at navigating rough terrains, such as cheetahs, cockroaches, and geckos., which will pave the path toward next-generation legged robots [152]. SLIDER robot achieves agile movement using linear hip motions, eliminating knee-hip rotations. Its lightweight design employs 5 DOFs per leg, totalling 10 DOFs, ensuring efficient and precise locomotion [147].
* Collaborative Systems
  Previously, legged robots were entirely controlled by computers or humans. They had no vision or sense of terrain height and no inertial sensing. As a result, behaving autonomously and intelligently in unpredictable and dynamically changing circumstances was impossible. Robots must also be able to identify instances when their objectives contradict the law that directs their actions. So they need to have a method of managing those circumstances with the help of collaborative systems. Human-robot interaction and robot-robot collaboration will significantly increase due to advancements in platforms, perception, and software power in the next generation of robots. These advancements would allow for the inclusion of various interactive and collaborative components in a growing environment of legged robotics. Next-generation legged robots are being developed thanks to collaborative systems, which combine human expertise with machine learning (ML) techniques. Robots can work together to accomplish tasks like navigating difficult terrain or carrying a heavy load. Additionally, collaborative systems make it easier to share data and knowledge, which boosts productivity, efficiency, and safety across a range of industries [153].
* Increased Efficiency, Reduced Cost, Suitability and Future Research
  Because of their difficulties navigating difficult terrain, confined spaces, and complex situations, legged robots have been a source of concern for the next generation. But as legged robots get more advanced, the goal is to increase their stability and control over their movements as well as have more energy-efficient locomotion [154]. Employing technologies like MQTT, power-efficient sensors and cloud planning optimizes energy use in swarm robots, considering future advancements. In addition to batteries, next-generation legged robots can use biological sources solar panels, wind energy, and hydrogen cells. However, these sources have limitations. Waste heat can also be converted into electricity using thermoelectric technology [143].

Despite this, existing legged robots still have useful characteristics for a variety of applications, including self-starting, lower cost, reduced vibration, and simple mechanisms. A research roadmap is therefore required to maximize the efficiency of legged robots and support their commercialization as more research opportunities exist in this area. Based on an examination of previous and recent advancements, this paper offers suggestions and directions for future studies on the control issues posed by legged robots. It discusses potential issues and their solutions while highlighting the necessity of developing future-legged robots capable of managing strategies in every imaginable field.

In order to enable legged robots to collaborate with humans and other robots in a variety of industries, future research in this area must continue to concentrate on enhancing the biomechanics of legged robots, creating more efficient control algorithms, and utilising collaborative systems.

Robots can achieve flexible movements and performance limits with optimization-based planning, but integrating it with online re-planning and locomotion is still difficult due to computational complexity. The computational complexity arises because real-time adjustments require rapid calculations that account for dynamic environmental changes, which can be resource-intensive. So, researchers are creating simpler models to streamline the optimisation process. Low-level locomotion algorithms aid in the robot's balance, whereas high-level algorithms improve autonomy by coordinating different locomotion technologies to meet different demands. The manipulation of the entire robot body may be necessary for legged robotics developments in the future to design collision-free trajectories in confined spaces. It is necessary to conduct research on the next generation of legged robots while taking into account the following requirements:

* Efficient and accurate external disturbance detection, estimation, and compensation with online re-planning based on optimization (e.g., to compensate for tracking, modelling, and estimation errors).
* Thorough planning that considers model uncertainties and reflexive and reactive tactics for obstacle avoidance and navigation in rough terrains.
* Task prioritization with model-based whole-body control.
* Planning non-periodic agile motions like jumping on high obstacles.
* Movement capability across deformable or constantly shifting terrain. Locomotion techniques may adapt to either gradual (such as grass, sand, mud, or gravel) or rapid changes in the landscape (e.g., rolling stones).
* Efficient patrolling, watching, and inspecting (e.g., acoustics, gas detection, radiation sensors).
* Moving effortlessly through confined spaces and crowded areas entails research on whole-body controllers and planners that use various contacts (for instance, in body parts other than the hands and feet) and prevent collisions with the surroundings.
* Benchmarking datasets and model terrain for generating suitable locomotion algorithms.

## 9 Conclusion

This study gives a detailed assessment of important technologies for legged locomotion, focusing on the implementation of kinematically and dynamically feasible motions that involve intricate interactions between robot feet and their environment. These technologies are critical for the creation of functional multi-legged robots that can navigate difficult situations with agility and stability. Their flexibility while moving should be increased, as should their terrain adaptation. The advantages of legged robots come with the challenge of complex control algorithms, some of which are difficult or impractical to implement effectively. So, this research provides a novel control approach model and mechanism to enhance biomechanical quality, boost energy efficiency, and add collaborative systems while improving control efficiency and decreasing the requirement for optimization. The literature review demonstrates that numerical trajectory optimization is typically used to treat alternating contacts and associated constraints on contact forces, but alternative control mechanisms, such as the linear inverted pendulum (LIP) model and other leg-in-the-loop models, may offer more efficient processing schemes [155]. The findings of this research show that future developments in legged robot control technology will allow for the use of sophisticated, but delicate, hardware to accomplish accurate movement and feedback. Furthermore, the control for legged balance analysis yielded consistent feedback, which can be done at a particularly low update frequency for both humanoid and quadruped robots. Our research shows that the effective implementation of a control approach is dependent on high-quality sensors with deformable components. An examination of the relevant literature indicates that the present control strategy for robot legs is based on sensors, data, and actuation. In the future, feedback mechanisms in robotic legs are expected to reach the precision of human legs. This advancement will enable their use in industrial operations and other critical tasks. It is feasible to rapidly change developing future technologies based on present components by using existing technologies such as artificial intelligence (AI)-based control and management, edge device-based cloud, digital twin models, smart elastic actuators, and enhanced networking.

After a brief review of the key necessities for legged robots, the literature presents the difficulties and potential for the next generation of limber robots based on several technological features. The Internet of Things (IoT), the Internet of Robotic Things (IoRT), communication protocols for robot-robot interactions, intelligent robotic legs, and supply chain management are a few of these. Electrical factors like power and energy efficiency are another. Together with operational considerations like task optimization, movement control, and quick response, computing issues including machine learning (ML), deep reinforcement learning (DRL), cyber connection, edge computing, swarm technologies, and AI are also included. The article then evaluates the long-term financial advantages for both manufacturers and consumers. The study explains the route toward deploying next-generation legged robots for future applications based on the appraisal of many ongoing research and technological advancements. Due to the movement control method's self-starting capability, high responsiveness to impediments, reliability, and simplicity of use, it is projected that it will become a popular choice for robot control mechanism framework extraction. Several of its fundamental flaws, such as the ineffectiveness in presenting the response time of a control system based on a high-quality sensor when passing barriers, have been fixed by recent developments. This paper provides a comprehensive analysis of the legged robot control method research trends and proposes a future research direction. The current work provides a research plan for legged locomotion control techniques. The following are the main findings of this study:

* Firstly, efforts in this area recently have concentrated on modifying control technique profiles and adding power-augmenting regulation devices. Moreover, different models have been applied to enhance the control of techniques on deformable terrains, especially at high speeds.
* Secondly, complex control strategies that can modify leg responses to impediments have been put forth. Maintaining a straightforward design and an adaptive control system that can recognize landscape elements implicitly is crucial.
* Thirdly, it is envisaged that AI-dominated design methodologies will offer a more precise method of movement direction prediction and a more appropriate model representation for managing legged mobility in the future. The proposed management approach will be easier and less expensive to test in many scenarios.
* Fourthly, to manage less tabulated data sets, feature engineering or cross-validation with highly productive controller approaches might be used. Also, by converting tabular data into visuals and applying better iterations of deep learning and highly trained fuzzy neural network methods for more effective action management, researchers can boost data size.

The study comes to the conclusion that it is now necessary to develop cutting-edge robot movement control techniques that can integrate multidisciplinary activities from all pertinent fields to achieve seamless coordination and functionality. To make it simpler to use robots in group activities, society's ideals and conventions should be taken into consideration when creating new robots.

## Additional information

No additional information is available for this paper.

## CRediT authorship contribution statement

**Swapnil Saha Kotha:** Writing – original draft, Methodology, Investigation, Formal analysis. **Nipa Akter:** Writing – original draft, Resources, Methodology, Investigation, Formal analysis. **Sarafat Hussain Abhi:** Writing – review & editing, Visualization, Validation, Supervision, Resources, Investigation, Conceptualization. **Sajal Kumar Das:** Writing – review & editing, Supervision. **Md. Robiul Islam:** Writing – review & editing, Validation. **Md. Firoj Ali:** Writing – review & editing, Validation. **Md. Hafiz Ahamed:** Writing – review & editing, Validation. **Md. Manirul Islam:** Writing – review & editing, Validation. **Subrata Kumar Sarker:** Writing – review & editing, Validation. **Md. Faisal Rahman Badal:** Writing – review & editing, Validation, Supervision. **Prangon Das:** Writing – review & editing, Validation. **Zinat Tasneem:** Writing – review & editing, Validation, Supervision. **Md. Mehedi Hasan:** Writing – review & editing, Validation.

## Declaration of Competing Interest

The authors declare that they have no known competing financial interests or personal relationships that could have appeared to influence the work reported in this paper.

## Data Availability

No data was used for the research described in the article.
